# Aboriginal medicinal plants of Queensland: ethnopharmacological uses, species diversity, and biodiscovery pathways

**DOI:** 10.1186/s13002-022-00552-6

**Published:** 2022-08-10

**Authors:** Gerry Turpin, Edita Ritmejerytė, Joanne Jamie, Darren Crayn, Phurpa Wangchuk

**Affiliations:** 1 Tropical Indigenous Ethnobotany Centre, Australian Tropical Herbarium, James Cook University, Building E1, Cairns Campus, McGregor Road, Smithfield, QLD 4878 Australia; 2 Queensland Herbarium, Department of Environment and Science, Mount Coot-tha Botanical Gardens, Mount Coot-tha Road, Toowong, QLD 4066 Australia; 3 Centre for Molecular Therapeutics, Australian Institute of Tropical Health and Medicine, James Cook University, Building E4, Cairns Campus, McGregor Road, Smithfield, QLD 4878 Australia; 4 Australian Tropical Herbarium, James Cook University, Building E1, Cairns Campus, McGregor Road, Smithfield, QLD 4878 Australia; 5 Centre for Tropical Environmental Sustainability Science, James Cook University, PO Box 6811, Cairns, QLD 4870 Australia; 6 School of Natural Sciences, Faculty of Science and Engineering, Macquarie University, North Ryde, NSW 2109 Australia

**Keywords:** Aboriginal medicinal plants, Queensland, Ethnopharmacological uses, Species diversity, Biodiscovery pathways

## Abstract

**Background:**

Aboriginal peoples have occupied the island continent of Australia for millennia. Over 500 different clan groups or nations with distinctive cultures, beliefs, and languages have learnt to live sustainably and harmoniously with nature. They have developed an intimate and profound relationship with the environment, and their use of native plants in food and medicine is largely determined by the environment they lived in. Over 1511 plant species have been recorded as having been used medicinally in Australia. Most of these medicinal plants were recorded from the Aboriginal communities in Northern Territory, New South Wales, South Australia, and Western Australia. Not much has yet been reported on Aboriginal medicinal plants of Queensland. Therefore, the main aim of this review is to collect the literature on the medicinal plants used by Aboriginal peoples of Queensland and critically assess their ethnopharmacological uses.

**Methods:**

The information used in this review was collected from archival material and uploaded into the Tropical Indigenous Ethnobotany Centre (TIEC) database. Archival material included botanist’s journals/books and old hard copy books. Scientific names of the medicinal plant species were matched against the ‘World Flora Online Plant List’, and ‘Australian Plant Census’ for currently accepted species names to avoid repetition. An oral traditional medical knowledge obtained through interviewing traditional knowledge holders (entered in the TIEC database) has not been captured in this review to protect their knowledge.

**Results:**

This review identified 135 species of Queensland Aboriginal medicinal plants, which belong to 103 genera from 53 families, with Myrtaceae being the highest represented plant family. While trees represented the biggest habit, leaves were the most commonly used plant parts. Of 62 different diseases treated by the medicinal plants, highest number of plants are used for treating skin sores and infections. Few plants identified through this review can be found in other tropical countries but many of these medicinal plants are native to Australia. Many of these medicinal plants are also used as bush food by Aboriginal peoples.

**Conclusion:**

Through extensive literature review, we found that 135 medicinal plants native to Queensland are used for treating 62 different diseases, especially skin infections. Since these medicinal plants are also used as bush food and are rarely studied using the Western scientific protocols, there is a huge potential for bioprospecting and bush food industry.

## Introduction

Globally, Indigenous biocultural knowledge (IBK) is gaining increasing recognition for its potential value in contemporary biodiversity conservation, land management, and biodiscovery [1]. Despite recent scientific medical advancements, traditional medicines (TM) based on IBK have gained significant attention due to growing health care demands and are considered as a primary health care modality. The World Health Organisation (WHO) acknowledges the importance of TM and describes it as the ‘sum total of the knowledge, skill, and practices based on the theories, beliefs, and experiences indigenous to other cultures, whether explicable or not, used in the maintenance of health, as well as in the prevention, diagnosis, improvement or treatment of physical and mental illnesses’ [2]. Other terms used for traditional medicines are complimentary, alternative, integrative, and customary medicines.

The WHO uses the term Traditional and Complimentary Medicines (TCM) [2], and it considers TCM an important and often underestimated health resource, particularly in the prevention and management of lifestyle-related chronic diseases [2]. The WHO Traditional Medicine Strategy 2014–2023 focusses on developing norms, standards, and technical documents based on reliable information and data. These will be used to provide support to its Member States in providing TCM services and its integration into their health systems. Currently, more than 85–90% of the world’s population uses Indigenous medicines for primary health care and approximately 50,000–70,000 plant species are used in Indigenous medicines [3]. In India and China, 20% and 19% of the local flora is used for treating various disorders, respectively [4], and more than 25% of prescription drugs worldwide are derived from plants with many more synthetic drugs obtained from phytochemical precursors [3, 5].

Aboriginal peoples have occupied the island continent of Australia for millennia. Over 500 different clan groups or nations with distinctive cultures, beliefs, and languages have learnt to live harmoniously within the varied eco-floristic zones: wet tropics, savannahs, evergreen forests, shrublands, grasslands, and wetlands [6, 7]. While there are a large number of different tribal groups in these vastly different regions, the commonality of all these groups is their intimate and profound relationship with the environment and the use of their resources [8]. Their knowledge and use of native plants plays significant and multiple roles in their lives, providing people with resources to make food, medicine, narcotics, stimulants, adornments, ceremonial objects, weapons, clothing, shelter, tools, and artwork [9, 10]. This Aboriginal knowledge lore has evolved over thousands of years, using native flora, fauna, and abiotic materials [11]. Without a written language, knowledge is maintained and shared as Aboriginal lore, and the customs and stories have been transferred intergenerationally through songs, stories, dance, and art.

Despite the disruption of oral traditions and lore practices by colonial contact, there are still thousands of Aboriginal people who speak their traditional language and retain the knowledge, songs, and customs of their ancestors with English being the second or third language [12, 13]. Aboriginal ethnomedicinal knowledge is still widely used by various Aboriginal clan groups but the extent to which it is practised varies widely amongst communities across Australia and between urban and rural regions [14]. Aboriginal plant knowledge can be considered the oldest living pharmacopoeia, with many plants still being consumed as bush food and bush medicine within Aboriginal communities, especially in remote areas [15].

**Fig. 1:**
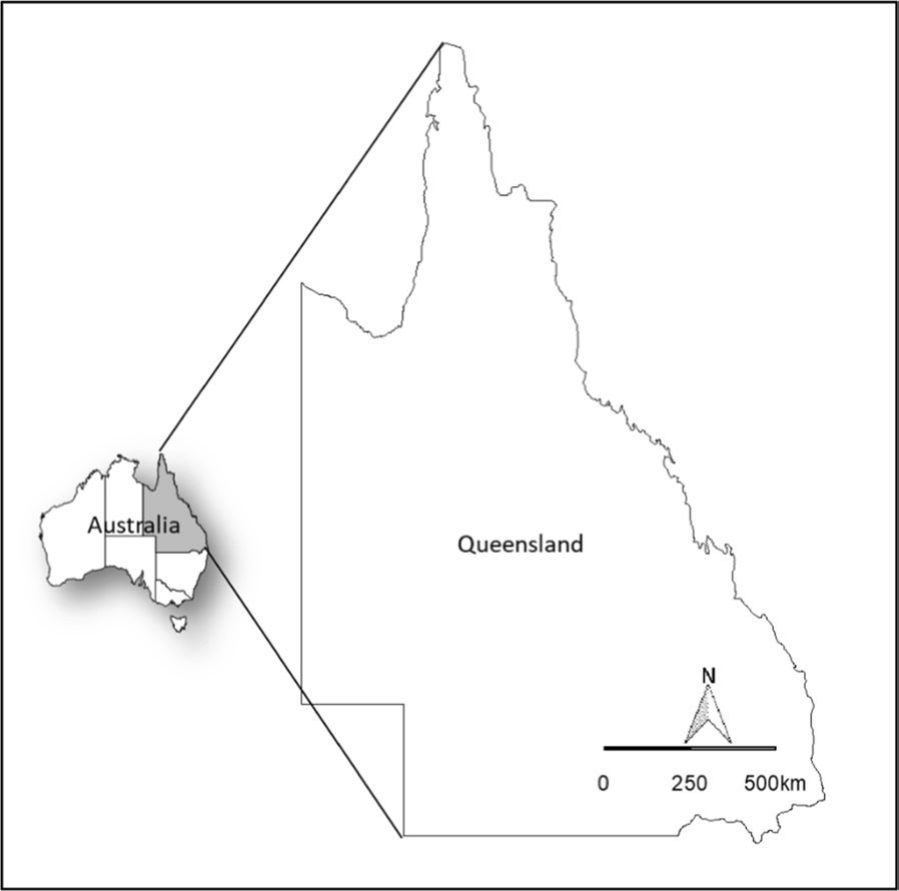
Map of Australia showing Queensland state—relevant to this review on medicinal plants

Overall, approximately 1511 plant species have been recorded as having been used medicinally in Australia [4, 15]. Most of the medicinal plant knowledge recorded in the literature belongs to the Aboriginal communities in Northern Territory, New South Wales, South Australia, and Western Australia [16]. Not much has yet been reported on Aboriginal medicinal plants of Queensland, with only limited publications on Aboriginal medicinal plants of Cape York Peninsula [17, 18]. There are still a large number of Aboriginal medicinal plants that are either unrecorded in the literature or not available in the public domain [16]. Much of this knowledge has been underexplored in a ‘Western scientific’ sense, and like much Indigenous knowledge worldwide, Australian Aboriginal knowledge is being eroded and is in critical danger of being lost forever [19]. This review describes and discusses 135 medicinal plants used customarily by Aboriginal people of Queensland (Fig. 1).

## Methods

The information used in this review was collected from archival material (uploaded into the Tropical Indigenous Ethnobotany Centre database—TIEC) [20], which mostly included journal papers and books. We also thoroughly searched the online databases and platforms such as Web of Science, Scopus, PubMed, and journal websites using the keywords such as: Aboriginal medicinal plants, Queensland medicinal plants, bush medicine, and bush food. A list of medicinal plants containing important information such as family, habit, parts used, and ethnopharmacological uses was tabulated. Scientific names of the medicinal plant species were compared against the ‘World Flora Online (WFO) Plant List’ [21] and ‘Australian Plant Census’ [22] for currently accepted species names to avoid repetition. Where the WFO and APC differ, we followed the latter. It is worth mentioning here that a number of medicinal plants that are not available in the public domain were excluded for this review as the Aboriginal communities want to protect their intellectual property rights. Since this review is on ethnobotanical aspects purely based on the information that is available online or in printed books, we did not include voucher specimen numbers and other scientific information such as phytochemical and pharmacological activities of medicinal plants.

## Results and discussion

### Historical perspectives and contribution of Aboriginal medicines

To put this review into overall Australian ethnomedical context, we analysed the literature to see what was the contribution of Aboriginal health system in Australia and worldwide. Historical studies and records of Aboriginal medicinal plants, e.g. [23–29], reveal their extensive use across Australia. While the medical practices of Aboriginal communities in Australia involve the use of plants [30, 31], there is little documentation of European use of this knowledge in early botanical texts. Stack [32] writes that 200 years ago, Aboriginal traditional medicine played no part in the lives of the European immigrants since they brought their own diseases and used their own traditional remedies. However, there are records, which show that during the early years of European settlement, some medical practitioners and botanists interacted with Aboriginal healers and experimented with native flora for medicinal purposes. For example, Denis Considen (1788–1794) (first assistant surgeon to Surgeon-General John White in the first fleet) claimed to be the first European medical practitioner to discover Indigenous medicinal plants; however, his methods of discovery are unknown and it is unclear if he involved Indigenous informants [33]. Considen documented some Indigenous medicinal plant efficacy including myrtle (possibly *Eugenia australis*) and yellow gum (possibly *Xanthorrhoea hastilis*) for dysentery, and native sarsaparilla (*Smilax glycyphylla*) as an antiscorbutic [33]. MacPherson [33] further suggested that native sarsaparilla was not only therapeutic but was considered more pleasant than Jamaican or Central American sarsaparilla. He also stated that prior to 1927 it had been a common article of trade among Sydney herbalists. One of the widely traded plants by both the Aboriginal people and the European settlers was macadamia nuts (*Macadamia* sp.).

Despite disruption of Aboriginal medical practices by Europeans, there have been some major contributions by Aboriginal Peoples to world medicinal knowledge. For example, Pearn [13] suggests that Aboriginal child care ethnomedicinal knowledge represents the ‘The world’s longest surviving paediatric practices’ [13]. Even today, Aboriginal ethnomedical practices form a living treasure trove of many Aboriginal communities. The use of medicinal plants by different communities across Australia is determined by the vegetation and environment they live in [14]. For example, the fruits of the native shrubs *Solanum laciniatum* are used in southern Australia, while *Solanum aviculare* (Kangaroo Apple) was used in the eastern parts of Australia [32]. Both species were used as poultices for joint swellings [32]. Both of these *Solanum* species contain an alkaloid solasodine, which is the precursor of cortisone and other steroids used in production of oral contraceptives (i.e. 'the pill') [34]. These plants have been imported into Russia and Eastern Europe where they are now cultivated at large scale, due to their capacity to biosynthesise this valuable phytochemical [32]. Similarly, the native Aboriginal narcotic shrub, pituri (*Duboisia hopwoodii)* of the arid interior region of Australia led to the research of congeneric, *Duboisia myoporoides*, which proved to be highly beneficial. Joseph Banks, the first European botanist to visit the east coast of Australia (in 1770), observed ‘pituri’ being chewed by Aboriginals similarly to tobacco or East Indian betele [35]. A century later (in 1872), Ferdinand von Mueller, the Victorian Government botanist, identified the plant as *Duboisia hopwoodii* [36]. Von Mueller suggested that the related *Duboisia myoporoides* should be researched, and it was found to produce hyoscine, currently known as scopolamine, an alkaloid that is a highly effective treatment for motion sickness [37].

### Queensland Aboriginal medicinal plants

**Fig. 2:**
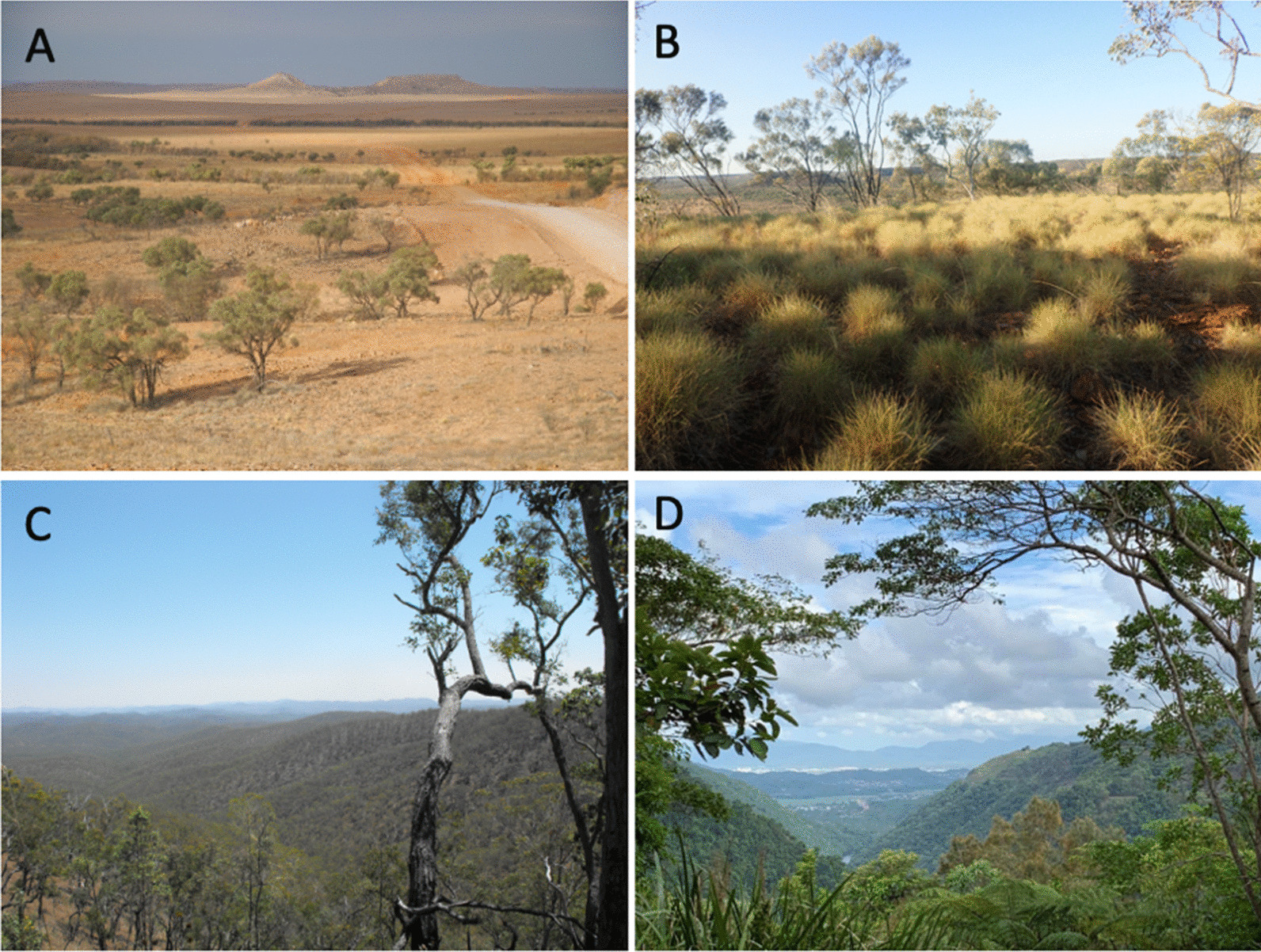
Representative vegetation of Queensland. **a** Desert. **b** Grassland. **c** Savannah. **d** Wet Tropics (Photo courtesy: Phurpa Wangchuk and Gerry Turpin)

Of all Australian States, Queensland, located in the north-east, encompasses the widest variety of landscapes, vegetation types, and climatic zones—temperate, wet and dry tropics, and semiarid to arid—across its 1.73 million square kilometres. The vegetation includes temperate, subtropical, and tropical rainforests, eucalypt woodlands, coastal communities and heath, a variety of grasslands and wetlands, and mangroves and marshes (Fig. 2) [38]. Queensland’s Aboriginal communities have lived in harmony within these landscapes for thousands of years. Their customary knowledge is shaped by the rich and diverse vegetation communities that are home to a diverse range of medicinal plant species, including that are endemic to the state. Some of the earliest records of medicinal plant knowledge in Queensland were made by botanists, chief protectors, anthropologists, pastoralists, and chemists [39]. For example, a pastoralist, amateur anthropologist, and politician Edward Palmer writes that Aboriginal people possess a considerable amount of knowledge of native plants and their uses [40].

Joseph Henry Maiden, who was the Curator of the Australian Museum at that time, wrote that Queensland is by far the richest of the colonies concerning recorded plants with medicinal properties [41]. However, he did not consult local Aboriginal people for medicinal plant knowledge stating that ‘In fairness to ourselves we must confess ourselves very little indebted to the Australian aboriginal for information as to the medical (or in fact any other) properties of our plants’ (Maiden, 1889). He also suggested that the great majority of these will be common to India and the Archipelago and have been employed by the natives of those countries [41]. Based on the little available literature and the oral medical traditions still practised by the rural Aboriginal elders and traditional knowledge holders of Queensland, it is fair to say that Maiden’s information on Aboriginal medical knowledge was limited and his perceptions were grossly misleading. Walter E. Roth, as Northern Protector of Aboriginals on Cape York Peninsula, recorded extensive information on Aboriginal customs, languages, and knowledge [42]. Similarly, Sandyl Kyriazis and Nai Beguta Agama Aboriginal Corporation have listed many medicinal plants in their book ‘Bush Medicine of the Northern Peninsula area of Cape York’ [43]. Leonard James Webb accompanied a team of anthropologists and Aboriginal people to document Indigenous plant uses at Lockhart River in northern Queensland between 1952 and 1977. He recognized the value of collaboration between scientists, anthropologists, and Indigenous peoples, combining the collection of scientific data with cultural perspectives in the study of plant uses [26, 44]. The inventory list (Table 1) supports the view that Queensland is rich in Aboriginal medicinal knowledge and plant diversity.

**Table 1:** List of Queensland medicinal plants and their uses [24, 25, 27, 32, 44–46]

Botanical name	Family	Habit	Part used	Diseases treated
*Abrus precatorius* L.	Fabaceae	Climber	Seeds	Abortion
*Acacia bivenosa* DC.	Fabaceae	Shrub	Bark, ash	Headache, colds, and fever
*Acacia holosericea* A.Cunn. ex G.Don	Fabaceae	Shrub	Root	Headache, colds, and fever
*Acacia melanoxylon* R.Br.	Fabaceae	Tree	Bark	Headache, colds, and fever
*Acalypha wilkesiana* Müll.Arg.	Euphorbiaceae	Shrub	Shoot	Sores/skin lesions/wounds/cuts
*Acmella grandiflora* (Turcz.) R.K.Jansen	Asteraceae	Herb	Root	Toothache
*Aegiceras corniculatum* (L.) Blanco	Primulaceae	Tree	Leaves, juice of leaves	Earache
*Ageratum conyzoides* L. subsp. *conyzoides*	Asteraceae	Herb	Whole plant	Sores/skin lesions/wounds/cuts
*Alphitonia excelsa* (A.Cunn. ex Fenzl) Benth.	Rhamnaceae	Tree	Bark, wood, young leaves, leaves, roots	Headache, colds, fever, stomach upset, snake bite, body ache, muscle pain, eye sores, skin lesions, wounds, cuts, toothache, diarrhoea, tonic
*Alphitonia petriei* Braid & C.T.White	Rhamnaceae	Tree	Bark	Body pain
*Alstonia constricta* F.Muell	Apocynaceae	Tree	Latex, bark	Skin infection, fever, tonic
*Alstonia scholaris* (L.) R.Br	Apocynaceae	Tree	Juice, sap, bark	Neuralgia, toothache, fever, sores, skin lesions, wounds, and cuts
*Alyxia spicata* R.Br.	Apocynaceae	Shrub	Root	Headache, breathlessness, colds, and fever
*Amyema quandang* (Lindl.) Tiegh	Loranthaceae	Hemi-parasitic shrub	Leaves	Fever, headache, and colds
*Antidesma bunius* (L.) Spreng	Phyllanthaceae	Tree	Fruit	Colds, fever, and headache
*Arivela viscosa* (L.) Raf	Cleomaceae	Herb	Whole plant, seeds, leaves	Colds and fever
*Asteromyrtus symphyocarpa* (F.Muell.) Craven	Myrtaceae	Shrub	Leaves	Colds, fever, and headache
*Barringtonia calyptrata* (Miers) R.Br. ex F.M.Bailey	Lecythidaceae	Tree	Leaves	Fever and chest pain
*Barringtonia racemosa* (L.) Spreng.	Lecythidaceae	Tree	Bark	Tonic
*Basilicum polystachyon* (L.) Moench	Lamiaceae	Herb	Whole plant	Fever
*Blainvillea acmella* (L.) Philipson	Asteraceae	Herb	Inner bark, fruit, root	Muscle sprain, bone aches, dislocation, broken bones
*Boerhavia diffusa* L.	Nyctaginaceae	Herb	Whole plant	Asthma
*Breynia cernua* (Poir.) Müll.Arg	Phyllanthaceae	Shrub	Leaves	Eye soreness
*Brucea javanica* (L.) Merr	Simaroubaceae	Shrub	Leaves, roots	Pain
*Buchanania obovata* Engl	Anacardiaceae	Tree	Inner bark, sapwood, leaves	Eye sores and toothache
*Calamus caryotoides* A.Cunn. ex Mart	Arecaceae	Palm	Shoot	Headache
*Callicarpa longifolia* Lam	Lamiaceae	Tree	Bark	Body pain
*Calophyllum inophyllum* L.	Clusiaceae	Tree	Kernel, fruit	Body pain and purgative
*Canarium australasicum* (F.M.Bailey) Leenh	Burseraceae	Tree	Bark	Stomach ache and diarrhoea
*Capparis lanceolaris* DC	Capparaceae	Climber	Bark	Sores, skin lesions, wounds, and cuts
*Capparis mitchellii* Lindl	Capparaceae	Tree	Bark	Sores, skin lesions, wounds, and cuts
*Carica papaya* L.	Caricaceae	Tree	Fruit	Prickly heat
*Cassytha filiformis* L.	Lauraceae	Climber	Macerated plant	Tonic
*Cassytha glabella* R.Br	Lauraceae	Climber	Whole plant, bark, leaves	Colds, fever, body pain, headache, sores, skin lesions, wounds, and cuts
*Casuarina equisetifolia* L.	Casuarinaceae	Tree	Inner bark	Toothache
*Centipeda thespidioides* F.Muell	Asteraceae	Herb	Whole plant	Sprains
*Cissus hypoglauca* A.Gray	Vitaceae	Climber	Fruit	Headache, colds, and fever
*Clematis glycinoides* DC	Ranunculaceae	Climber	Leaves	Headache, colds, and fever
*Clematis microphylla* DC	Ranunculaceae	Climber	Whole plant, seeds, leaves	Headache, colds, and fever
*Clerodendrum floribundum* R.Br	Lamiaceae	Tree	Wood	Pain
*Clerodendrum inerme* (L.) Gaertn	Lamiaceae	Shrub	Leaves, roots	Sores, skin lesions, wounds, cuts, and sprains
*Cocos nucifera* L.	Arecaceae	Tree	Oil, bark, coconut jelly	Earache, swelling, sores, skin lesions, wounds, and cuts
*Coelospermum decipiens* Baill	Rubiaceae	Shrub	Leaves, roots	Pregnancy prevention
*Convolvulus erubescens* Sims	Convolvulaceae	Herb	Whole plant	Diarrhoea
*Corymbia gummifera* (Gaertn.) K.D.Hill & L.A.S.Johnson	Myrtaceae	Tree	Leaves, gum	Bleeding control, diarrhoea, ringworm, and sexually transmitted diseases
*Corymbia polycarpa* (F.Muell.) K.D.Hill & L.A.S.Johnson	Myrtaceae	Tree	Kino	Toothache and dysentery
*Corymbia terminalis* (F.Muell.) K.D.Hill & L.A.S.Johnson	Myrtaceae	Tree	Bark	Dysentery
*Corymbia tessellaris* (F.Muell.) K.D.Hill & L.A.S.Johnson	Myrtaceae	Tree	Gum	Constipation
*Crinum pedunculatum* R.Br	Amaryllidaceae	Herb	Whole plant	Stings (marine)
*Crotalaria cunninghamii* R.Br	Fabaceae	Shrub	Leaves, sap, bark	Pain
*Croton arnhemicus* Müll.Arg	Euphorbiaceae	Tree	Root	Sores, skin lesions, wounds, cuts, and stomach ache
*Cyathea australis* (R.Br.) Domin	Cyatheaceae	Tree fern	Young leaves	Stomach ache and tonic
*Cymbidium canaliculatum* R.Br	Orchidaceae	Orchid	Bulb	Dysentery
*Cymbidium madidum* Lindl	Orchidaceae	Orchid	Bulb, seeds	Dysentery, pregnancy prevention
*Cymbonotus lawsonianus* Gaudich	Asteraceae	Herb	Leaves	Sores, skin lesions, wounds, and cuts
*Cymbopogon ambiguus* (Hack.) A.Camus	Poaceae	Grass	Leaves	Colds
*Cymbopogon bombycinus* (R.Br.) Domin	Poaceae	Grass	Whole plant	Eye soreness
*Cymbopogon obtectus* S.T.Blake	Poaceae	Grass	Whole plant	Eye soreness
*Cymbopogon* sp*.*	Poaceae	Grass	Root	Earache
*Cynanchum viminale* subsp. *australe* (R.Br.) Meve & Liede	Apocynaceae	Succulent shrub	Latex, tuber, sap	Sores, skin lesions, wounds, cuts, warts, and gonorrhoea
*Deplanchea tetraphylla* (R.Br.) F.Muell	Sapotaceae	Tree	Bark	Colds and influenza
*Derris* sp.	Fabaceae	Climber	Bark	Sores, skin lesions, wounds, and cuts
*Dioscorea transversa* R.Br	Dioscoreaceae	Climber	Tuber	Skin cancer
*Dodonaea polyandra* Merr. & L.M.Perry	Sapindaceae	Tree	Root	Sores, skin lesions, wounds, cuts, and toothache
*Erythrophleum chlorostachys* (F.Muell.) Baill	Fabaceae	Tree	Bark	Sores, skin lesions, wounds, cuts, pain, and sprain
*Eucalyptus haemastoma* Sm	Myrtaceae	Tree	Kino	Diarrhoea, wounds, ulcers
*Eucalyptus pruinosa* Schauer	Myrtaceae	Tree	Bark	Rheumatism and body pain
*Eucalyptus resinifera* Sm	Myrtaceae	Tree	Leaves, inner bark, gum	Ringworm
*Eucalyptus tetrodonta* F.Muell	Myrtaceae	Tree	Leaves	Fever and headache
*Euphorbia tirucalli* L.	Euphorbiaceae	Succulent shrub	Latex	Skin cancer
*Euphorbia mitchelliana* Boiss	Euphorbiaceae	Shrub	Flowers	Diarrhoea
*Excoecaria agallocha* L.	Euphorbiaceae	Mangrove tree	Latex	Stings (marine)
*Excoecaria parvifolia* Müll.Arg	Euphorbiaceae	Tree	Bark	Body pain
*Exocarpos aphyllus* R.Br	Santalaceae	Succulent shrub	Bark, roots	Boils
*Ficus fraseri* G.Forst	Moraceae	Tree	Milky juice of young roots	Sores, skin lesions, wounds, and cuts
*Ficus microcarpa* L.f	Moraceae	Tree	White sap	Stings (fish)
*Ficus opposita* Miq	Moraceae	Tree	Latex, leaves, gum	Sores, skin lesions, wounds, cuts, and fungal infections including ringworm
*Flagellaria indica* L.	Flagellariaceae	Climber	Fresh new growth tips	Sores, skin lesions, wounds, cuts, and pox
*Flueggea virosa* (Willd.) Voigt	Phyllanthaceae	Shrub	Root	Toothache
*Grevillea mcgillivrayi* I.M.Turner	Proteaceae	Tree	Leaves	Sore throat
*Grevillea striata* R.Br	Proteaceae	Tree	Charcoal, bark	Sores, skin lesions, wounds, cuts, hives, and stings
*Grewia retusifolia* Kurz	Malvaceae	Shrub	Inner bark of roots, roots, leaves, fruits	Diarrhoea, dysentery, boils, swelling, toothache, stomach ache, sores, skin lesions, wounds, cuts, and cough
*Haemodorum corymbosum* Vahl	Haemodoraceae	Herb	Root	Constipation
*Heliotropium ovalifolium* Forssk	Boraginaceae	Herb	Extract	Body wash and fever
*Hibiscus vitifolius* L.	Malvaceae	Herb	Tuber	Boils
*Ipomoea pes-caprae* (L.) R.Br	Convolvulaceae	Climber	Whole plant, leaves, stems	Sexually transmitted diseases, boils, and swelling
*Litsea glutinosa* (Lour.) C.B.Rob	Lauraceae	Tree	Leaves, bark	Sores, skin infection, lesions, wounds, cuts, eye sores, body pain, scabies, gastritis, fever, headache, influenza, and paediatric uses
*Macaranga tanarius* (L.) Müll.Arg	Rubiaceae	Tree	Red sap	Sores, skin lesions, wounds, and cuts
*Manihot esculenta* Crantz	Euphorbiaceae	Shrub	Root	Diarrhoea and stomach ache
*Melaleuca leucadendra* (L.) L.	Myrtaceae	Tree	Young leaves, bark	Cough and cold, headache, tonic, sinusitis, sores, skin lesions, wounds, and cuts
*Melaleuca quinquenervia* (Cav.) S.T.Blake	Myrtaceae	Tree	Young leaves	Cold, headache, and tonic
*Melaleuca viridiflora* Sol. ex Gaertn	Myrtaceae	Tree	Leaves	Cough
*Melicope vitiflora* (F.Muell.) T.G.Hartley	Rutaceae	Tree	Juice, resin, gum, bark	Toothache, bodyache, and toothache
*Mentha australis* R.Br	Lamiaceae	Herb	Whole plant	Cough and cold
*Morinda citrifolia* L.	Rubiaceae	Tree	Fruit	Cough and cold, sore throat
*Mucuna gigantea* (Willd.) DC	Fabaceae	Climber	Seed	Colds, fever, headache
*Musa banksia* F.Muell	Musaceae	Tree	Sap, juice	Stings (stinging tree), paralysis and headache
*Myristica globosa* subsp*. muelleri* (Warb.) W.J.de Wilde	Myristicaceae	Tree	Gum from bark	Ringworm
*Nauclea orientalis* (L.) L.	Rubiaceae	Tree	Bark	Rheumatism, colds, stomach ache, and snake bite
*Ocimum tenuiflorum* L.	Lamiaceae	Herb	Leaves, stems	Influenza, labour pain, and stomach ache
*Pandanus* sp*.*	Pandanaceae	Palm-like	Base of leaf	Body pain, sore throat, sores, skin lesions, wounds, and cuts
*Pandanus spiralis* R.Br	Pandanaceae	Palm-like	Sap, base of leaf	Sores, skin lesions, wounds, and cuts
*Persicaria subsessilis* (R.Br.) K.L.Wilson	Polygonaceae	Herb	Whole	Sores, skin lesions, wounds, and cuts
*Persoonia falcata* R.Br	Proteaceae	Shrub	Leaves, bark, wood	Cough and cold, sore throat, and eye sores
*Petalostigma pubescens* Domin	Picrodendraceae	Shrub	Fruit, root	Toothache
*Phyllanthus urinaria* L	Phyllanthaceae	Herb	Leaves	Colds
*Piper hederaceum* (Miq.) C.DC	Piperaceae	Climber	Whole plant	Sore gums
*Planchonella pohlmaniana* (F.Muell.) Pierre ex Dubard	Sapotaceae	Tree	Twigs and leaves	Boils
*Planchonia careya* (F.Muell.) R.Knuth	Lecythidaceae	Tree	Bark, leaves	Tonic and body pain
*Plectranthus congestus* R.Br	Lamiaceae	Herb	Leaves and branches	Body pain and syphilis
*Plectranthus parviflorus* Willd	Lamiaceae	Herb	Leaves	Syphilis
*Plumeria rubra* L.	Apocynaceae	Tree	Leaves	Swelling
*Pseudognaphalium luteoalbum* (L.) Hilliard & B.L.Burtt	Asteraceae	Herb	Whole plant	Fever and tonic
*Psidium guajava* L.	Myrtaceae	Tree	Fruit	Constipation and stomach ache
*Pterocaulon serrulatum* (Montrouz.) Guillaumin	Asteraceae	Herb	Leaves	Chest congestion, fever, colds, headache, sores, skin lesions, wounds, and cuts
*Ripogonum album* R.Br	Ripogonaceae	Climber	Bark, roots	Stings (stingray)
*Santalum lanceolatum* R.Br	Santalaceae	Tree	Scraped outer wood, inner moist bark, leaves	Chest ailments, purgative, sexually transmitted diseases, arthritis, insect bites, sores, skin lesions, wounds, and cuts
*Santalum obtusifolium* R.Br	Santalaceae	Shrub	Wood	Constipation and pain
*Scoparia dulcis* L.	Plantaginaceae	Herb	Whole plant	Influenza, sores, stomach ache, skin lesions, wounds, and cuts
*Senna alata* (L.) Roxb	Fabaceae	Shrub	Leaves	Fungal infections specially ringworm, heat rash, scabies, and skin itches
*Stemodia viscosa* Roxb	Plantaginaceae	Herb	Whole plant	Body pain and tonic
*Sterculia quadrifida* R.Br	Sterculiaceae	Tree	Leaves, branches, barks, roots	Eye sores, skin lesions, wounds, cuts, and nausea
*Striga curviflora* (R.Br.) Benth	Linderniaceae	Herb	Whole plant	Sores, skin lesions, wounds, and cuts
*Syzygium suborbiculare* (Benth.) T.G.Hartley & L.M.Perry	Myrtaceae	Tree	Bark, roots	Body pain
*Tabernaemontana orientalis* R.Br	Apocynaceae	Shrub	Rootbark, sap	Fever, sores, skin lesions, wounds, and cuts
*Tephrosia turpinii* Pedley	Fabaceae	Herb	Tuber	Sores, skin lesions, wounds, and cuts
*Tephrosia varians* (F.M.Bailey) C.T.White	Fabaceae	Herb	Root	Sores, skin lesions, wounds, and cuts
*Terminalia catappa* L	Combretaceae	Tree	Bark, young green fruit	Sore throat and thrush
*Terminalia muelleri* Benth	Combretaceae	Tree	Soft leaves	Scabies, sores, skin lesions, wounds, and cuts
*Tetrameles nudiflora* R.Br	Datiscaceae	Tree	Leaves	Swelling, sores, skin lesions, wounds, and cuts
*Timonius timon* (Spreng.) Merr	Rubiaceae	Tree	Inner bark	Colds, fever, influenza
*Tribulus cistoides* L.	Menispermaceae	Climber	Whole plant	Toothache
*Wrightia saligna* (R.Br.) F.Muell. ex Benth	Apocynaceae	Tree	Inner bark, milky sap, root	Boils, sores, skin lesions, wounds, and cuts
*Xenostegia tridentata* (L.) D.F.Austin & Staples	Convolvulaceae	Climber	Whole plant	Sores, skin lesions, wounds, and cuts
*Xylomelum scottianum* (F.Muell.) F.Muell	Proteaceae	Tree	Leaves, roots	Body pain

### Inventory of Queensland Aboriginal medicinal plants

**Fig. 3:**
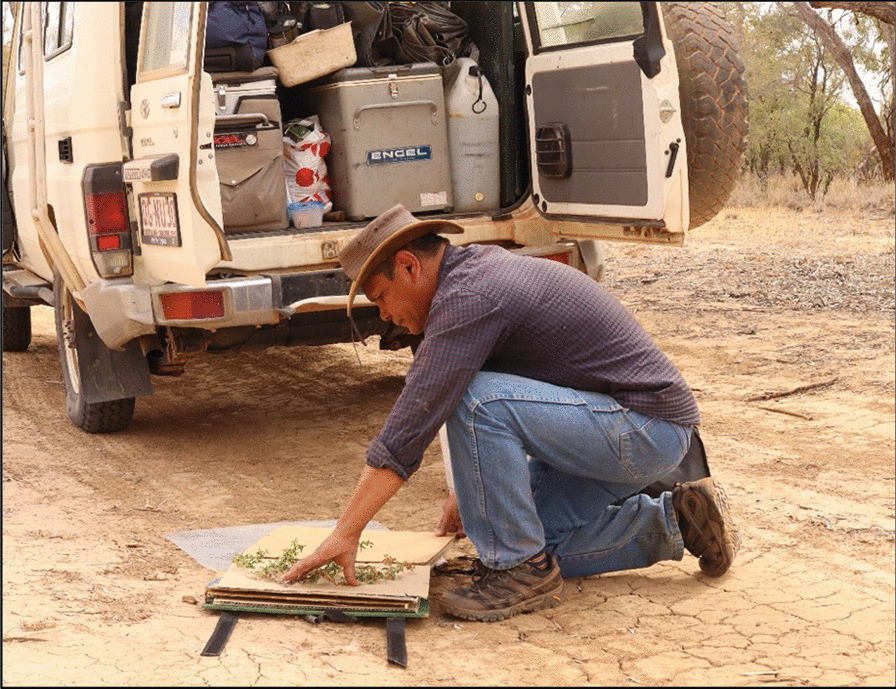
An Indigenous ethnobotanist Gerry Turpin collecting herbarium specimens of Aboriginal medicinal plants (Photo courtesy—Gerry Turpin, first author of this manuscript)

Based on the archival information from the TIEC database (maintained by an Indigenous ethnobotanist Mr. Gerry Turpin, Fig. 3) and the online information, we have identified a total of 135 species of medicinal plants used by Queensland Aboriginal people. The archival information is presented in Table 1, including botanical name, plant family, habit, plant part used, and diseases/conditions treated.

### Diversity of medicinal plants

Table 1 lists the botanical names of 135 species of plants used customarily as medicines by Aboriginal people of Queensland. These plants belong to three plant groups—angiosperms, gymnosperms, and pteridophytes—and represent a total of 53 families and 103 genera. Plant families with most species with recorded medicinal properties were Myrtaceae (14), Fabaceae (11), Lamiaceae (8), and Apocynaceae, Asteraceae, and Euphorbiaceae (7 each). It is no surprise that Myrtaceae is recorded as having the most medicinal uses of any plant family as it is one of the most common but largely tropical families worldwide, estimated to include about 5,950 species in about 132 genera [47]. Queensland has 60 genera and 746 species of Myrtaceae [48]. Many plants within the Myrtaceae family contain oils in the leaves, and oil is one of the best solvents for medicinal compounds. Within the Myrtaceae family, there are five genera used medicinally in Queensland: *Eucalyptus* (seven species used medicinally), *Melaleuca* (four species), *Corymbia* (two species), *Syzygium* (one species) and the introduced *Psidium guajava*. *Corymbia* and *Eucalyptus* are two of the three genera (the other being *Angophora*) that comprise the ‘eucalypts’, a group of iconic Australian forest trees including more than 800 species that together dominate 77% of Australia’s native forests [49]. While fungi, seaweed, and bryophytes (liverworts, hornworts, and mosses) were commonly used globally as foods and medicines, the record of use, particularly in Queensland, is very poor [45, 50]. Lichens were also not recorded.

### Habits of Queensland medicinal plants

**Fig. 4:**
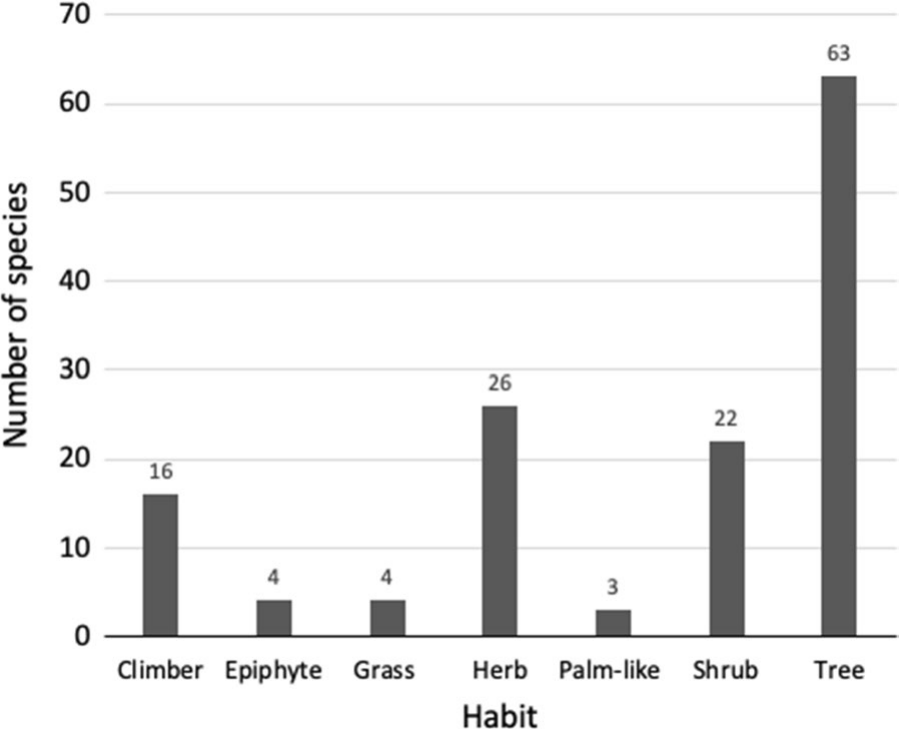
Category of habits of medicinal plants. **a** Bar graph showing number of species in each habit category

Of the 135 species of Queensland medicinal plants (Table 1), 62 species are trees, followed by herbs (26 species), shrubs (22 species), climbers (16 species), epiphytes and grasses (4 species each), and palm-like (3 species) (Fig. 4). The epiphyte category includes orchids and hemiparasitic mistletoes; shrubs include small erect, woody plants, and succulent shrubberies; and trees include mangroves and tree ferns.

### Plant parts used for treating diseases

**Fig. 5:**
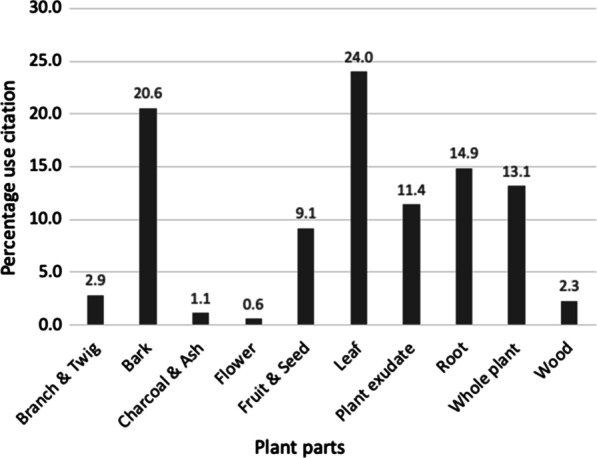
Category of medicinal plants parts and their percentage of uses citation

Over millennia, Aboriginal people have determined which plant parts are useful medicinally. Table 1 shows different parts of 135 medicinal plants grouped into 10 major categories: ‘Branch & Twig’, ‘Bark’, ‘Charcoal & Ash’, ‘Flower’, ‘Fruit & Seed’, ‘Leaf’, ‘Plant exudate’, ‘Root’, ‘Whole plant’ and ‘Wood’ (Fig. 5). The category ‘Bark’ includes outer bark, inner cambium, and root bark; ‘Fruit & Seed’ comprises fruit, seed, and kernel; ‘Leaf’ includes young leaf, leaf stalk, leaf tip, and young shoot; ‘Plant exudate’ includes oil, sap, gum, resin, kino, and latex; and ‘Root’ includes tap root, tuber, and bulb. The category ‘Whole plant’ is mostly smaller herbaceous plants and vines where it was more productive and easier to collect the roots, stems, and leaves together rather than separating plant parts. Of 10 major plant part categories, ‘Leaf’ ranked first in terms of percentage use with 24%, followed by bark (20.6%), root (14.9%), whole plant (13.1%), plant exudate (11.4%), fruit & seed (9.1%), branch & twig (2.9%), wood (2.3%), charcoal & ash (1.1%), and flower (0.6%), for example, the leaves of *Aegiceras corniculatum*, the barks of *Acacia melanoxylon*, the roots of *Flueggea virosa*, *Alphitonia excelsa* as whole plant, *Corymbia polycarpa* for kino (plant exudate), the fruits of *Morinda citrifolia*, branches and twigs of *Cassytha filiformis*, the wood of *Clerodendrum floribundum*, and the flowers of *Euphorbia mitchelliana*. It is to be expected that leaves show the highest amount of usage in Aboriginal medicine, given that leaves are in abundance, easily accessed and processed. Leaves are also readily harvested typically without much damage to the plant and readily replenished, making their use sustainable. Leaves are an easy target not only for humans but also for herbivores.

### Types of diseases treated by medicinal plants

A total of 62 types of diseases are treated by 135 medicinal plants listed in Table 1. Most medicinal plants are used for treating disease of the nervous system, integumentary system, respiratory system, and digestive system. Over 17% of these medicinal plants have been reported to be used for treating ‘Skin sores and infections’ (integumentary system), followed by ‘Cuts and wounds’ (12.6%), ‘Stomach disorders’ (digestive system) and ‘Pain’ (nervous system) (8.5% each), ‘Fever’ (7.9%), ‘Cough and cold’ (respiratory system) (7.6%), ‘Headache’ (6.3%), ‘Toothache’ (3.8%), ‘Eyesore’ (3.8%), and ‘Sexually transmitted disease’ (1.9%). Interestingly, 3.2% of plants were recorded for their use as tonics. For example, *Cassytha filiformis* and *Barringtonia racemosa* are described as good health tonics for the body. The biggest disease category ‘Skin sores and infections’ includes sores, boils, ringworm, hives, skin irritation, and other fungal and bacterial infections. Similarly, the category ‘Stomach disorders’ comprises stomach ache, gastritis, constipation, diarrhoea, and dysentery. The disease category ‘Pain’ includes body ache/pain, earache, muscle pain, labour pain, and chest pain; the ‘Sexually transmitted diseases’ consist of gonorrhoea, syphilis, and other infections.

Of 135 medicinal plant species, 53 of them have been cited for the treatment of a single disease (one plant–one disease treatment category). For example, *Acmella grandiflora* and *Euphorbia tirucalli* are used for treating only toothache and skin cancer, respectively. Similarly, while *Coelospermum decipiens* is used as a contraceptive, *Abrus precatorius* is used for abortion. However, the majority of medicinal plants have been used for treating more than one disease. For example, *Alphitonia petriei* is used for treating headache, cough and cold, fever, stomach upset, body ache, muscle pain, snake bite, eye sore, skin lesion, cuts/wounds, toothache, diarrhoea and as a tonic. Another plant, *Grewia retusifolia*, is used for treating diarrhoea, dysentery, boils, swelling, toothache, stomach ache, sores, skin lesions, wounds, cuts, and cough. Four plant species are used for treating stings from marine organisms such as rays, fishes, and sea jellies.

For treating the diseases, decoctions are commonly used in herbal medicine and these are preparations from plants using water. While decoctions, especially the tonics, are frequently ingested for diseases such as gastritis, cough and cold, and fever, poultices are commonly used for external skin diseases. Some medicinal plants are chewed and swallowed. There are a few plant species that are burnt and used as fumigants. Some plant products are used as infusions.

### Biodiscovery initiatives involving Queensland medicinal plants

Biodiscovery involves identification of novel drug lead compounds from various natural resources such as plants, animals, fungi, bacteria, and extremophiles. Novel and effective drugs can be developed from natural products, especially from medicinal plants using a biorational ethnobotany-guided strategy. Indeed, out of 122 current plant-derived prescription drugs, 80% were discovered from medicinal plants [3]. Well-known examples are quinine (antimalarial compound isolated from *Cinchona officinalis*), artemisinin (antimalarial compound isolated from *Artemisia annua*), paclitaxel (anticancer compound isolated from *Taxus baccata*), vincristine (anticancer compound isolated from *Catharanthus rosea*), aspirin (anti-inflammatory compound derived from salicylic acid isolated from *Salix babylonica*), and morphine (analgesic compound isolated from *Papaver somniferum*). Rainforest plants are the source of a quarter of pharmaceutical products, and more than 70% of these plant species are found exclusively in the tropical Amazon rainforest [51].

Queensland harbours the great majority of Australia’s tropical rainforest, which represents a warehouse of the continent’s medicinal treasure trove [52]. However, the biodiversity of these rainforests, along with other species-rich tropical biomes such as the Great Barrier Reef, is under threat of climate change [53–55]. Consequently, the changes in the environment might trigger plant physiological responses as well as adaptations in secondary metabolism to produce either higher concentrations or novel phytochemicals to cope with abiotic stress (Mounter, 2019). It is well known that such anti-stress biomolecules exhibit potent antioxidant and anti-inflammatory properties [56–59], which have potential applications in novel drug development.

The rich and diverse vegetation of Queensland has shaped the development of unique ethnobotanical knowledge of Aboriginal and Torres Strait Islander people, which has huge potential to guide biodiscovery programmes. Early investigations of Aboriginal medicinal plant knowledge in Queensland were carried out by chemists and pharmacologists in the nineteenth century. For example, Joseph Bancroft analysed properties of the pituri narcotic used by inland Aboriginal groups, which was later discovered to contain nicotine alkaloids [60, 61]. Commencing in 1944, an Australian Phytochemical Survey was established by Leonard James Webb at the Commonwealth Scientific and Industrial Research Organisation (CSIRO) in Brisbane, but few Queensland medicinal plants were screened for their phytochemicals [44, 62]. In 1984, CSIRO staff based in Melbourne began publishing the results of the Phytochemical Survey [29]. However, it does not contain comprehensive information on bioactive chemical constituents of Queensland medicinal plants.

A recent review by Janice Mani and colleagues [63] reported the antioxidative and therapeutic potential of selected number of Australian plants, which included only a limited number of Queensland medicinal plants. While some medicinal plants that are distributed globally may have been studied for their phytochemical and pharmacological activities, there are hundreds of Queensland native plants that remain unexplored for therapeutic applications. Indeed, Australian medicinal plants in general remain underexplored in terms of biological, medicinal, and economic resources. Given that the globally popular natural medicines and their related products from other parts of the world (worth an estimated US$83 billion [64, 65]) has created lucrative marketplaces and that the Australian agriproducts are already enjoying an international reputation for their high-quality and clean image, there are exclusive demands for the Queensland medicinal plants. More elaborate studies are therefore required to develop quality parameters to monitor the quality of medicinal plants (both cultivated and wild type) and to identify biomarker and bioactive compounds. This will not only improve our knowledge on the phytochemistry of Queensland medicinal flora, but also generate data and ideas for developing herbal industries related to health-promoting, pharmaceutical, nutraceuticals, cosmetics, and functional food products. This has a huge scope for the development of a sustainable regional development of Queensland, Indigenous workforce development, and promotion of plant-based biotechnological innovations both locally and worldwide.

### Biodiscovery frameworks, community engagement, and research approaches

In the past, especially in the 1970s–1990s, biopiracy has commonly occurred and the innovations and intellectual property rights belonging to many Indigenous peoples around the world have been exploited and compromised by the researchers, institutions, companies, and pharmaceutical industries [66]. This was partly due the lack of proper protocols, ethical rules and regulations, and biodiscovery acts. It has left Indigenous peoples dry and void of their rights, which has partly led Indigenous communities to step back from collaborating with the researchers and companies. In order to investigate the biocultural knowledge of medicinal plants used by Indigenous communities, consents and approvals from Traditional Owners/corporations must be adequately addressed [11]. At times, negotiations and signing collaborative/benefit sharing agreement with the communities can take longer, which will impede the access to plant materials and progress of research. This partly explains the relative scarcity of documented ethnomedicinal knowledge about plants used by the Australian Aboriginal and Torres Strait Islander peoples, especially in the biodiscovery space. However, if the biodiscovery research project is built upon the trust and the long-term relationship of the parties, this gap can be bridged easily.

We found that it is important to conduct any bioprospecting/biodiscovery projects in line with global ethical guidelines and intellectual property rights practices advocated by the following national and international bylaws and regulations.

- World Intellectual Property Organization (relevant section on Indigenous knowledge) [67].
- Nagoya Protocol on Access and Benefit-sharing and Traditional Knowledge [68].
- NHMRC ethical guidelines for research with Aboriginal and Torres Strait Islander Peoples [69].
- The AIATSIS Code of Ethics for Aboriginal and Torres Strait Islander Research [70].
- Queensland Biodiscovery Act 2004 on using traditional knowledge for biodiscovery, which provides step-wise traditional knowledge guidelines and biodiscovery resources tool kits [71].
- Relevant university’s research code of conduct such as James Cook University Aboriginal and Torres Strait Islander Research Ethics [72].

The World Health Organisation realizes the importance of traditional medicines both as the source of health care and novel drug leads for modern medicine, and therefore their long-term survival and sustainability is imperative. Australia’s Biodiversity Conservation Strategy (2010–2030) has acknowledged that preservation and sustainable maintenance of Indigenous knowledge is a priority area and therefore it is essential to actively engage Indigenous people through employment, partnership, and transfer of scientific knowledge that actively supports its sustainable use.

#### Building community relationships and engagement

It is important to develop collegial relationship with the Indigenous communities and propose the project to the funding bodies together as a team. The community members should be involved as a partner in shaping the research activities and the project must facilitate two-way exchange of knowledge, skills, and benefits and should provide capability building opportunities. Informed consent and ethics from the Indigenous community must be obtained prior to any information collection or documentation or product development based on traditional knowledge. It is also imperative to sign proper memorandum of understandings or collaborative agreement or benefit sharing agreement with the relevant Indigenous community. To build better trust and relationships, researchers must identify the cultural broker (Indigenous background) and provide the community with: (a) results from each aim of the project on a yearly basis, (b) study tours to research stations, (c) community researchers’ seminars where both sides present their ideas, challenges, and needs, (d) staying connected with the community, and (d) co-authorship on relevant publications. During engagement with the communities, the National Indigenous Science Education Program developed by Jamie and her group at Macquarie University can be adapted to improve the enrolment of Indigenous people in the higher education sector and guide them to become scientists, health workers, and policy changers.

#### Benefits to the Indigenous communities

The project design should find the needs/benefits/requirements of the Indigenous communities prior to biodiscovery grant applications. The benefits to the Indigenous community would include: (a) community development support fees to help the community in conducting administrative and legal duties relevant to the projects, (b) employment opportunities, (c) training the Indigenous communities on preservation of customary medical knowledge, collection and cultivation of quality medicinal plants, and intellectual property rights protection, d) transfer of skills and knowledge to enable the community to carry on similar biodiscovery initiatives on their own even after this project has ended, (e) developing commercial products for their own use and marketing, and (f) fair distribution or share of the income from the sale of their new drug lead molecules to the pharma companies. Many Indigenous communities in Queensland live in remote areas with limited access to conventional modern medicine, which are available in larger towns and cities. These remote communities still use their culturally accustomed plants as bush food and bush medicines. Therefore, the biodiscovery projects must generate ethically sound scientific data to support their uses by the communities, while at the same time discover novel drug leads with commercial prospects. In addition, the toxicity data on medicinal plants should help the Indigenous communities to make more informed decisions on the safe use of those medicinal plants. Achieving these objectives will add values, confidence and pride to their cultural identity and will improve the preservation and sustainability of their health care knowledge, and also that of the medicinal biota of the rainforests. To ensure that the precious first-hand customary knowledge about medicinal biota is preserved and promoted for future generations, the community-authored bush medicine handbooks and a passworded online database on Customary Medicinal Knowledgebase must be supported through the projects.

## Conclusion and future directions

This review identified 135 species of Queensland Aboriginal medicinal plants, which belong to 103 genera from 53 families, with Myrtaceae being the highest represented plant family. While trees represented the biggest habit, leaves were the most commonly used plant parts. Of 62 different diseases treated by the medicinal plants, a large number of plants are used for treating skin sores and infections. Few plants identified through this review can be found in other tropical countries, but many of these medicinal plants are native to Australia. Many of these native medicinal plants are rarely studied for their phytochemical and pharmacological properties and have a huge potential for discovering novel drug lead compounds.

Therefore, there is an urgent need to study the biota with these unexplored medicinal plants for developing novel plant-based drugs. However, it is vital that the biodiscovery projects should benefit the Indigenous communities in Queensland fairly and equitably in accordance with the international and national biodiscovery bylaws and regulations. Prior to any biodiscovery project proposals, it is recommended that the Indigenous communities are consulted first and engaged in the beginning as collaborators/partners and equal decision-makers for how grants are applied for if the biodiscovery project funding is limited to only laboratory experiments and consumables (which is the case in most of the project funds), the research organizations should explore means to help the collaborating communities with the community development support services. It is necessary that we build this awareness so that research groups can apply for this funding and ensure that collaboration provides value to all the participants in a project.

## Data Availability

Data sharing is not applicable to this article as no data sets were generated or analysed during the current study.
